# Endoscope-Assisted Spine Surgery: A Comprehensive Review of Clinical Applications and a Lateral Interbody Fusion Case Illustration

**DOI:** 10.7759/cureus.86600

**Published:** 2025-06-23

**Authors:** Brandon M Edelbach, Jeffrey Lubisich, Vadim Gospodarev, Rasha Elbadry, Namath Hussain

**Affiliations:** 1 Neurological Surgery, Loma Linda University School of Medicine, Loma Linda, USA; 2 Neurosurgery, Loma Linda University School of Medicine, Loma Linda, USA; 3 Neurological Surgery, Loma Linda University Medical Center, Loma Linda, USA; 4 Neurosurgery, Loma Linda University Medical Center, Loma Linda, USA

**Keywords:** endoscopic approach, endoscopic discectomy, lumbar-fusion, minimially invasive surgery, spinal decompression, spine microsurgery

## Abstract

Endoscopic spine surgery offers a minimally invasive alternative to traditional microsurgical techniques across a spectrum of pathologies, namely discectomy, interbody fusion, and laminectomy, by leveraging small incisions, reduced tissue disruption, and direct visualization. Endoscopic discectomy yields shorter operative times, lower infection rates, and comparable pain and functional outcomes versus open microdiscectomy. Similarly, endoscopic laminectomy for lumbar stenosis provides equivalent decompression with less postoperative pain and faster mobilization than microscopic or open approaches. In the realm of interbody fusion, endoscopic techniques (including transforaminal lumbar interbody fusion, lateral lumbar interbody fusion, and extreme lateral interbody fusion (XLIF) variants) achieve fusion rates and radiographic corrections on par with minimally invasive surgical (MIS) techniques, while further minimizing blood loss, length of stay, and anesthesia exposure. We present a 67-year-old male patient with prior T10-11 and T11-12 fusions, hemilaminectomies, and dorsal column stimulator hardware who developed adjacent-segment L3-4 stenosis. An endoscopic XLIF was performed under fluoroscopic and neuromonitoring guidance. The patient experienced an uncomplicated procedure with immediate preservation of neurologic function and postoperative imaging confirming ideal cage placement and alignment. Endoscopic spine surgery, exemplified by this XLIF case, combines the benefits of MIS with an expanded view of the surgical field. With proper patient selection and adherence to evolving technical guidelines, these techniques continue to offer promising outcomes, reinforcing their role as a transformative modality in modern spine surgery.

## Introduction

Traditionally, spinal decompression, reconstruction, and stabilization procedures have been performed via open surgery, which, while effective, are often associated with significant tissue disruption, increased postoperative pain, longer hospital stays, and extended recovery periods. Over the past several decades, the advent of minimally invasive spine surgery (MIS) has revolutionized the field of spine surgery, largely in parallel with advances in technology. The popularization of microscopic surgical techniques in the 1960s by pioneers such as Yasargil facilitated the introduction of microsurgical approaches to the spine resulting in reduction in incision size, thereby reducing tissue damage and enhancing visualization of the operative field [1]. Building on these foundational techniques, the integration of endoscopic methods into spine surgery emerged in the 1980s, initially through the adaptation of percutaneous indirect spinal canal decompression techniques [2,3].

A significant milestone in this evolution was the development of the transforaminal endoscopic approach. Dr. Parviz Kambin described an anatomical corridor located at the posterolateral aspect of the lumbar intervertebral foramen. This triangle is delineated by the superior endplate of the lower vertebral body, the superior articular process, and the inferior aspect of the exiting nerve root, providing a “safe zone” for instrument passage during endoscopic procedures [2]. Subsequent studies have further refined this approach utilizing foraminotomy to expand the surgical window through the anterior aspect of the superior articular process. These initial advancements have led to development of a wide variety of endoscopic approaches [4-7].

The incorporation of endoscopic techniques into MIS represents a further refinement of spine surgery, with benefits including further reduction in incision sizes, minimization of tissue disruption, and enhanced operative precision [8-10]. As these innovations continue to evolve, they not only improve patient outcomes through decreased postoperative morbidity and faster recoveries but also redefine the standards of safety and efficacy in spinal care.

## Case presentation

A 67-year-old man with a history of lumbar fusion, T10-11 and T11-12 left hemilaminectomies, and dorsal column stimulator paddle lead placement presented for progressive lower extremity pain and motor weakness. MRI of the lumbar spine demonstrated L3-4 degenerative disk disease and adjacent segment disease with central stenosis. Given the patient’s history of multiple prior posterior surgeries, including hemilaminectomies and dorsal column stimulator placement, an anterior or lateral approach was preferred to minimize disruption of scarred posterior elements. Extreme lateral interbody fusion (XLIF) was selected for its ability to achieve adequate decompression and alignment correction through a minimally invasive lateral corridor. The patient was taken to the operating room (OR) for L3-4 lateral interbody fusion with a plate.

Patient positioning

**Figure 1:**
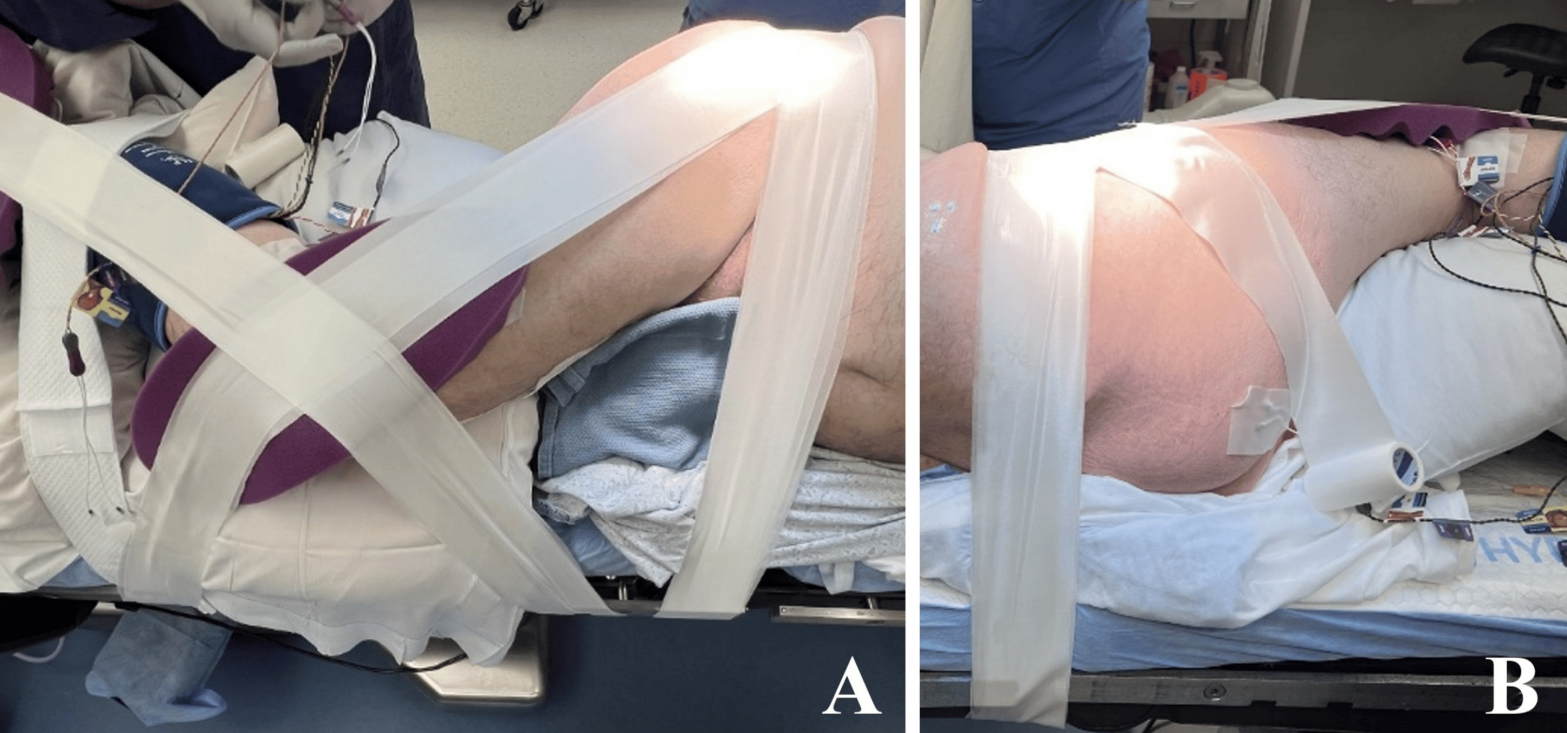
Patient Positioning Patient in right lateral decubitus position on table with adhesive tape providing traction on the lower extremities (A) and pelvis (B) and the table “broken” in the lumbar region to introduce lateral flexion, optimizing the XLIF surgical corridor. XLIF: Extreme lateral interbody fusion

The patient was then positioned in the right lateral decubitus. All extremities were carefully padded, an axillary roll was placed, and pillows were positioned between the arms and knees. Tape was run from the pelvis to the foot of the bed, around the foot of the bed, and back up to the pelvis. Additional tape was run from the pelvis to the foot of the bed, and the table was broken at the lumbar region to laterally flex the spine to optimize the anticipated retroperitoneal approach as illustrated in Figure 1.

Initial approach

To facilitate safe access to the retroperitoneal space while protecting the peritoneum, an oblique incision was made at the primary surgical site for direct dissection, followed by a second posteriorly located incision to allow for tactile guidance and displacement of the peritoneum during the lateral approach. Following incision of the skin, dissection through the subcutaneous tissues was performed and Weitlaner retractors were placed in the incision site to aid in dissection. Lateral entrance of the retroperitoneal space was achieved through careful dissection and palpation of the internal oblique and transversus abdominis layers by placing the surgeon’s finger in the second posterior incision. These muscle layers were transversed while concurrently using a finger to sweep away any at risk peritoneum. Upon completion of dissection, palpation of the iliopsoas muscle and the transverse process of the vertebral body was achieved.

Discectomy and interbody fusion

**Figure 2:**
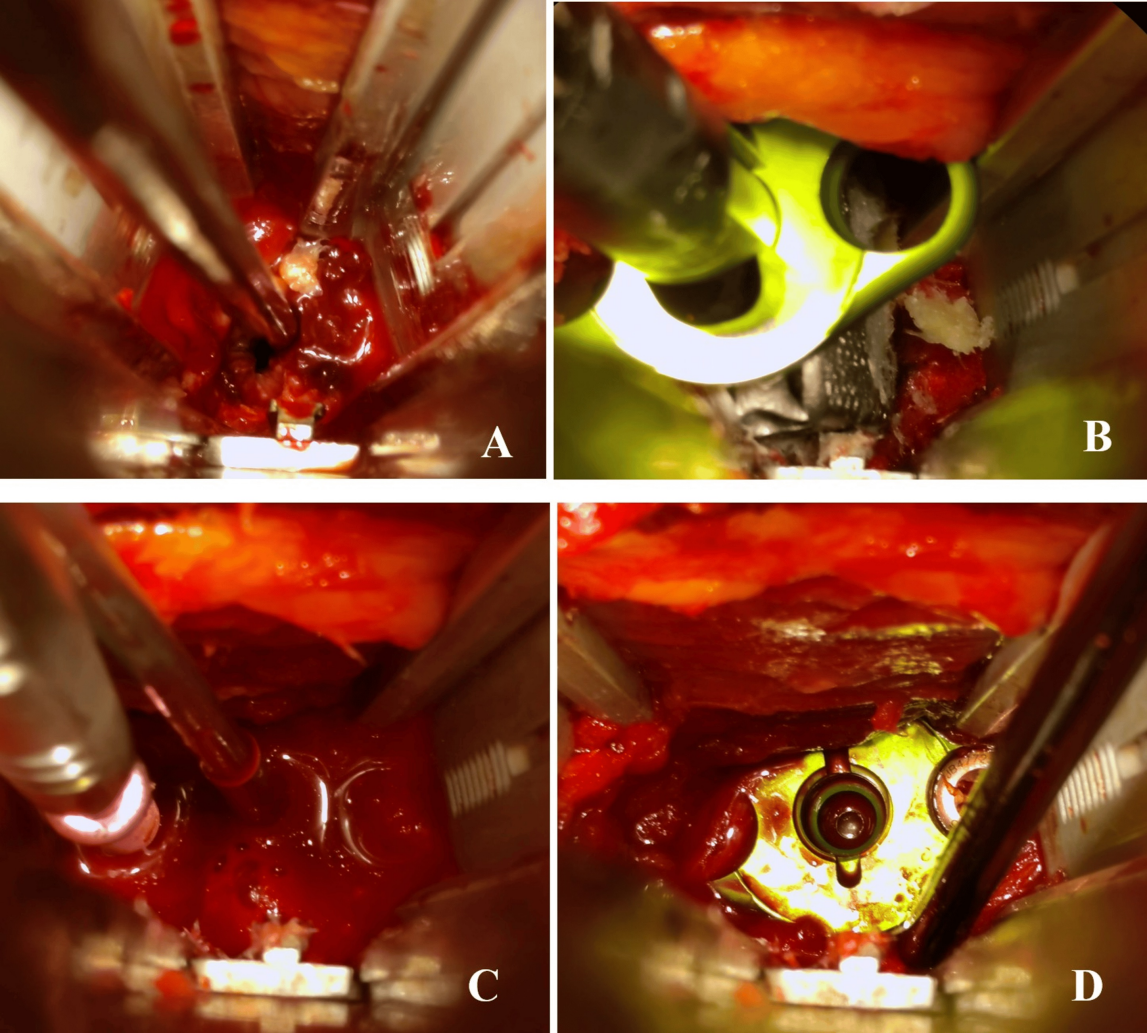
Endoscopic Visualization of the Interbody Spacer. Endoscopic view (A) discectomy, (B) placement of the interbody spacer, (C) screw placement, and (D) final interbody space positioning.

**Figure 3:**
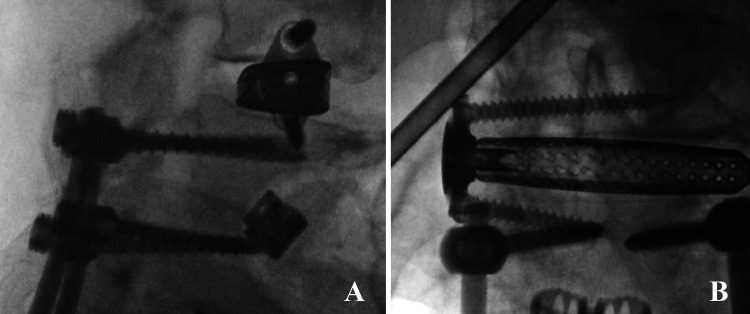
Lateral (A) and Anterior-posterior (B) Lumbar X-ray Demonstrating Placement of the L3-4 Interbody Spacer. Postoperative lateral lumbar x-ray demonstrating final placement of L3-4 interbody spacer.

Having completed dissection, a small tubular retractor was advanced from the lateral incision to the surface of the iliopsoas muscle over the L3-4 disk space. After positioning was verified with fluoroscopy, the tubular retractor was advanced through the iliopsoas muscle with neuromonitoring. Visualization was achieved using an endoscope. A retractor was placed and soft tissue was cleared from the lateral edge of the disc and the annulus was excised (Figure 2A). Discectomy, autograft harvesting, and placement of an intervertebral spacer were completed under endoscopic visualization (Figures 2B-2D). Proper placement of the cage and alignment were verified using intraoperative fluoroscopy (Figure 3).

Outcome

The estimated blood loss was approximately 25 mL, and the total operative time was 97 minutes. The patient experienced an uncomplicated postoperative course with preserved neurologic function and no immediate complications. He was discharged on postoperative day two with oral analgesics which were tapered over a month course. The patient was followed for six months and reported improvement in pain control.

## Discussion

Interbody fusion

XLIF is a lateral retroperitoneal, trans-psoas approach to the lumbar spine commonly utilized for interbody fusion, corpectomies, and deformity corrections [11]. XLIF was devised to reduce vascular injury and muscular trauma associated with the transforaminal, posterior, and posterolateral lumbar fusion approaches. However, XLIF carries a unique risk of neurological injuries, particularly to nerves of the lumbar plexus [12]. XLIF is limited to T6-L5 levels and may be challenging or unsafe at L4-L5, given the angle of instrumentation caused by obstruction of the iliac crest [13] and close relationship of the lumbar plexus and intervertebral disk [14]. In the thoracic spine, the aorta and anatomical variations of the azygos system of veins pose a risk [12,15,16]. Bowel perforation, though infrequent, is a serious complication associated with XLIF, which, if not promptly identified, may lead to severe morbidity [12]. Incidence of bowel injury appears to be higher when the L2-L3 levels are involved and with greater numbers of fused segments [17]. At the L1-L4 levels, injury of the iliohypogastric, ilioinguinal, and lateral femoral cutaneous nerves may occur during dissection as these nerves travel obliquely, inferiorly, and anteriorly in the retroperitoneal space to reach the iliac crest and abdominal wall [12,18,19]. Splitting the psoas major at L3 or L4 poses a risk to the genitofemoral nerve as it travels obliquely and anteriorly within the psoas muscle from its origin, emerging superficially along the anterior surface of the psoas [11,19]. The lumbosacral plexus, positioned most dorsally at L1-L2, migrates progressively ventrally, shrinking the safe operating window with each descending root level to L4-L5 [11]. Hence, posterior dilator or retractor placement may result in nerve injury, and L4-L5 procedures pose the greatest risk to the plexus [11,12]. While the incidence of major vascular injuries is low in XLIF, the retroperitoneal greater vessels and lumbar arteries also pose a notable risk [12,20]. The greater vessels are of particular risk at the L4/L5 levels as they migrate posteriorly to varying degrees before bifurcation occurs [11,12,15]. This risk of injury is heightened in patients with degenerative scoliosis as these vessels are further posteriorly positioned, resulting in an even smaller safe operative window [20,21].

To mitigate these risks, detailed pre-operative radiological planning is necessary to identify anatomical variations [12,15]. To avoid neurological injury, intraoperative techniques such as real-time electromyographic monitoring and fluoroscopic guidance are commonly employed in XLIF. Interoperative monitoring modalities, including finger electrodes, motor evoked potentials, the use of no muscle relaxants, and “triggered” EMGs, have also been utilized to limit nerve injury [12,22]. Despite advancements in monitoring and guidance, the limited direct visualization of critical structures remains a significant challenge in XLIF procedures. Identification and repair of injury to at-risk retroperitoneal vessels can also be difficult, given the small operating window. The novel integration of an interoperative camera may offer enhanced visualization of these at-risk structures, enabling a tighter operative window and reducing the risk of complications. Furthermore, improved visualization should allow for more aggressive dissection while also facilitating the prompt identification and repair of damage during XLIF procedures.

The most widely described endoscopic lateral interbody fusion (LIF) procedures are the TLIF, LLIF, and XLIF (Table 1). Meta-analyses comparing endoscopic TLIF to MIS-TLIF demonstrated significantly shorter recovery times and earlier post-operative pain relief associated with endoscopic TLIF [23-25]. These results were supported in a clinical trial conducted in China comparing endoscopic TLIF to MIS-TLIF. Furthermore, the Chinese study found no difference between the correction of segmental lumbar lordosis and overall lumbar lordosis between the two cohorts [26]. Additionally, a meta-analysis by Li et al. compared unilateral biportal endoscopic LIF and endoscopic LIF. The study found that unilateral biportal endoscopic LIF had shorter operative times and reduced learning curve, while endoscopic LIF had less total blood loss and shorter hospitalization times. Both techniques showed comparable outcomes in terms of postoperative pain, complications, and fusion rates [25,27]. Zhuo et al. compared two-segment lumbar endoscopic LIF to MIS-LIF and found similar results to single-segment studies [28].

**Table 1:** Comparison of common minimally invasive and endoscopic spine surgery techniques Description of common lateral minimally invasive and endoscopic approaches, to spine including common indications and limitations.

Technique	Approach	Common Indications	Key Advantages	Limitations
XLIF (Extreme Lateral Interbody Fusion)	Lateral retroperitoneal, trans-psoas	Lumbar degenerative disease, deformity, adjacent segment disease	Large interbody grafts, minimal posterior disruption, good deformity correction	Risk to lumbar plexus, limited access at L4–5
TLIF (Transforaminal Lumbar Interbody Fusion)	Posterolateral	Lumbar instability, degenerative disc disease, spondylolisthesis	Posterior approach, avoids anterior vascular structures	More muscle disruption, limited disc space access
PEID (Percutaneous Endoscopic Interlaminar Discectomy)	Posterior interlaminar endoscopic	L5–S1 disc herniations	Shorter operative time, minimally invasive, reduced paraspinal muscle damage	Limited to lower lumbar levels
PETD (Percutaneous Endoscopic Transforaminal Discectomy)	Posterolateral transforaminal endoscopic	Foraminal and extraforaminal disc herniations	Minimally invasive, less paraspinal muscle damage	Risk to exiting nerve root
UBE (Unilateral Biportal Endoscopy)	Posterior biportal	Decompression, discectomy, interbody fusion	Enhanced visualization	Multiple access ports necessary

Discectomy

One of the earliest applications of endoscopic techniques in spine surgery was discectomy, as documented by Kambin and Gellman via the aforementioned posterolateral transforaminal approach [2,29]. This approach is suitable for a variety of disc herniations, including contained, migrated, foraminal, and extraforaminal herniations [4].

Overall, comparisons of percutaneous endoscopic lumbar discectomy (PELD) and open lumbar microdiscectomy (OLM) for lumbar disc herniation consistently demonstrate that PELD is associated with reduced operative times, length of hospital stays, and quicker return to work [5,8-10,30-33]. While many authors found that PELD to be associated with reduced postoperative pain, statistically significant differences in VAS and ODI scores between PELD and OLM were not consistently reported [8,10,34]. Similarly, differences in complication, recurrence, and reoperation rates were not consistently statistically different when compared by several recent meta-analyses [8,9,34,35]. In a nationwide retrospective study conducted in Korea, OLM was found to be associated with higher rates of post-operative infection. PELD has 16.8% infection rate, and OLM is associated with 23.9% infection rate. This was attributed to smaller operative corridors and shorter length of stay [36]. Chen et al. also noted a reduced infection rate associated with PELD but also noted inconsistency in reported rates of infection in the dataset and significant risk of publication bias associated with the result [32]. Lastly, Son et al. noted that PELD was associated with a significantly steeper learning curve when compared to OLM [37].

The percutaneous endoscopic lumbar transforaminal discectomy (PETD) and percutaneous endoscopic lumbar interlaminar discectomy (PEID) approaches are the dominant methodology for PELD. Nie et al. and Chen et al. performed prospective randomized controlled trials comparing PEID and PETD for L5-S1 disc herniation. They reported shorter operation times and less intraoperative radiation exposure for PEID, with no significant differences in postoperative bed rest time, hospitalization time, or complication rates. Both approaches showed similar improvements in ODI and VAS scores [38,39]. He et al. conducted a meta-analysis of randomized trials comparing PETD and PEID. Their results echoed that of the aforementioned trials, demonstrating that PEID had significantly shorter operation times and fewer fluoroscopy times, with similar postoperative outcomes [40].

Wang et al. and Yin et al. compared unilateral biportal endoscopic discectomy and PEID for high-grade down-migrated lumbar disc herniation and L5-S1 disc herniation respectively [41,42]. In both studies, PEID was associated with shorter operative duration, reduced intraoperative blood loss, and shorter hospital stays. Both techniques demonstrated similar clinical efficacy.

In addition to pure endoscopic approaches, combined micro-endoscopic approaches have been described. Chen et al. compared PETD and micro-endoscopic discectomy (MED) in a randomized controlled trial, publishing results at both the two and five-year follow-up [43,44]. Both techniques had comparable long-term clinical outcomes and recurrence rates, with PETD associated with worse results for median disc herniation, while MED was associated with worse results in lateral disc herniation. Meta-analysis on retrospective data also found comparable outcomes in terms of pain relief [5,6].

Laminectomy

When evaluating the performance of endoscopic approaches for decompression similar results have been described as those associated with discectomy. According to meta-analysis comparing endoscopic to microscopic laminectomy for lumbar stenosis, there was significantly lower post-operative pain and shorter length of hospital course but no significant difference in any other functional or surgical outcomes [45]. Of note, again in two meta-analyses, there was discordance in results on rates of complications between endoscopic and microsurgical laminectomy [46,47]. As these findings are not reflected in well-controlled randomized trials, it is likely that these differences stem from heterogeneity in smaller case series. Hwang et al. conducted a prospective trial to compare open, endoscopic and biportal endoscopic laminectomy for lumbar stenosis and found that endoscopic assisted laminectomy was associated with improved muscle preservation and post-operative pain when compared to open techniques [48]. Kotheeranurak et al. conducted a randomized controlled trial comparing endoscopic and tubular-assisted microscopic laminectomy for lumbar stenosis and found no difference in outcomes between the two approaches [49]. Similarly, studies comparing uniportal and biportal endoscopic laminectomy failed to demonstrate any significant differences [50].

Despite its advantages, endoscopic XLIF possesses notable limitations. It is generally not suitable in cases requiring direct decompression of the spinal canal, where posterior visualization is essential. Patients with significant posterior element pathology, including facet arthropathy or instability, may benefit more from posterior approaches. Additionally, access to the L4-L5 level may be obstructed by a high-riding iliac crest.

## Conclusions

Endoscopic spine surgery represents a transformative advancement in minimally invasive spinal interventions, offering a versatile alternative to traditional microsurgical techniques. Endoscopic approaches afford a targeted approach, permitting smaller incisions and reduced tissue disruption. Furthermore, endoscopic spinal approaches consistently demonstrate comparable functional outcomes to microsurgery in select spinal pathologies and have been associated with reduced operative times and faster postoperative recovery. The current evidence robustly supports their efficacy in treating a wide range of spinal pathologies. Ultimately, endoscopic spine surgery is reshaping modern spinal care, demonstrating promise in enhancing both efficiency and patient outcomes.

## References

[1] Yaşargil MG (1999). A legacy of microneurosurgery: memoirs, lessons, and axioms. Neurosurgery.

[2] Kambin P, Gellman H (1983). Percutaneous lateral discectomy of the lumbar spine a preliminary report. Clin Orthop Relat Res.

[3] Hijikata S (1989). Percutaneous nucleotomy. A new concept technique and 12 years' experience. Clin Orthop Relat Res.

[4] Kim HS, Paudel B, Jang JS, Lee K, Oh SH, Jang IT (2018). Percutaneous endoscopic lumbar discectomy for all types of lumbar disc herniations (LDH) including severely difficult and extremely difficult LDH cases. Pain Physician.

[5] Shi R, Wang F, Hong X (2019). Comparison of percutaneous endoscopic lumbar discectomy versus microendoscopic discectomy for the treatment of lumbar disc herniation: a meta-analysis. Int Orthop.

[6] Yu P, Qiang H, Zhou J, Huang P (2019). Percutaneous transforaminal endoscopic discectomy versus micro-endoscopic discectomy for lumbar disc herniation. Med Sci Monit.

[7] Ahn Y (2023). Anterior endoscopic cervical discectomy: surgical technique and literature review. Neurospine.

[8] Qin R, Liu B, Hao J, Zhou P, Yao Y, Zhang F, Chen X (2018). Percutaneous endoscopic lumbar discectomy versus posterior open lumbar microdiscectomy for the treatment of symptomatic lumbar disc herniation: a systemic review and meta-analysis. World Neurosurg.

[9] Jarebi M, Awaf A, Lefranc M, Peltier J (2021). A matched comparison of outcomes between percutaneous endoscopic lumbar discectomy and open lumbar microdiscectomy for the treatment of lumbar disc herniation: a 2-year retrospective cohort study. Spine J.

[10] Kim M, Lee S, Kim HS, Park S, Shim SY, Lim DJ (2018). A comparison of percutaneous endoscopic lumbar discectomy and open lumbar microdiscectomy for lumbar disc herniation in the Korean: a meta-analysis. Biomed Res Int.

[11] Arnold PM, Anderson KK, McGuire RA Jr (2012). The lateral transpsoas approach to the lumbar and thoracic spine: a review. Surg Neurol Int.

[12] Epstein NE (2019). Review of risks and complications of extreme lateral interbody fusion (XLIF). Surg Neurol Int.

[13] Quack V, Eschweiler J, Prechtel C (2022). L4/5 accessibility for extreme lateral interbody fusion (XLIF): a radiological study. J Orthop Surg Res.

[14] Guérin P, Obeid I, Bourghli A (2012). The lumbosacral plexus: anatomic considerations for minimally invasive retroperitoneal transpsoas approach. Surg Radiol Anat.

[15] Buric J, Bombardieri D (2016). Direct lesion and repair of a common iliac vein during XLIF approach. Eur Spine J.

[16] Koutsouflianiotis KN, Paraskevas GK, Kitsoulis P, Noussios G (2019). A case of abnormal origin of the Azygos vein system: an anatomical study in a human cadaver. Cureus.

[17] Hwang ES, Kim KJ, Lee CS (2022). Bowel injury and insidious pneumoperitoneum after lateral lumbar interbody fusion. Asian Spine J.

[18] Grunert P, Drazin D, Iwanaga J (2017). Injury to the lumbar plexus and its branches after lateral fusion procedures: a cadaver study. World Neurosurg.

[19] Uribe JS, Arredondo N, Dakwar E, Vale FL (2010). Defining the safe working zones using the minimally invasive lateral retroperitoneal transpsoas approach: an anatomical study. J Neurosurg Spine.

[20] Santillan A, Patsalides A, Gobin YP (2010). Endovascular embolization of iatrogenic lumbar artery pseudoaneurysm following extreme lateral interbody fusion (XLIF). Vasc Endovascular Surg.

[21] Assina R, Majmundar NJ, Herschman Y, Heary RF (2014). First report of major vascular injury due to lateral transpsoas approach leading to fatality. J Neurosurg Spine.

[22] Ozgur BM, Aryan HE, Pimenta L, Taylor WR (2006). Extreme lateral interbody fusion (XLIF): a novel surgical technique for anterior lumbar interbody fusion. Spine J.

[23] Kou Y, Chang J, Guan X, Chang Q, Feng H (2021). Endoscopic lumbar interbody fusion and minimally invasive transforaminal lumbar interbody fusion for the treatment of lumbar degenerative diseases: a systematic review and meta-analysis. World Neurosurg.

[24] Wang Q, Chang S, Dong JF, Fang X, Chen Y, Zhuo C (2023). Comparing the efficacy and complications of unilateral biportal endoscopic fusion versus minimally invasive fusion for lumbar degenerative diseases: a systematic review and mate-analysis. Eur Spine J.

[25] Li X, Qu Y, Zhou L, Zhou Y, Peng B, Duo J (2025). Meta-analysis of the clinical efficacy and safety of unilateral biportal endoscopic lumbar interbody fusion versus endoscopic lumbar interbody fusion for the treatment of lumbar degenerative diseases. World Neurosurg.

[26] Ma T, Tu X, Li J (2025). Comparative analysis of clinical efficacy of unilateral biportal endoscopic and open transforaminal lumbar interbody fusion in the treatment of lumbar degenerative. Front Surg.

[27] Ding Y, Chen H, Wu G, Xie T, Zhu L, Wang X (2024). Comparison of efficacy and safety between unilateral biportal endoscopic transforaminal lumbar interbody fusion versus uniportal endoscopic transforaminal lumbar interbody fusion for the treatment of lumbar degenerative diseases: a systematic review and meta-analysis. BMC Musculoskelet Disord.

[28] Zhuo C, Liu Y, Zhang Y (2024). Comparison of the short-term efficacy of MIS-TLIF and Endo-LIF for the treatment of two-segment lumbar degenerative disease. BMC Musculoskelet Disord.

[29] Telfeian AE, Veeravagu A, Oyelese AA, Gokaslan ZL (2016). A brief history of endoscopic spine surgery. Neurosurg Focus.

[30] Yu H, Zhu B, Liu X (2021). Comparison of percutaneous endoscopic lumbar discectomy and open lumbar discectomy in the treatment of adolescent lumbar disc herniation: a retrospective analysis. World Neurosurg.

[31] Song SK, Son S, Choi SW, Kim HK (2021). Comparison of the outcomes of percutaneous endoscopic interlaminar lumbar discectomy and open lumbar microdiscectomy at the L5-S1 level. Pain Physician.

[32] Chen X, Chamoli U, Vargas Castillo J, Ramakrishna VA, Diwan AD (2020). Complication rates of different discectomy techniques for symptomatic lumbar disc herniation: a systematic review and meta-analysis. Eur Spine J.

[33] Latka K, Kozlowska K, Domisiewicz K, Klepinowski T, Latka D (2025). Full-endoscopic lumbar spine discectomy: are we finally there? A meta-analysis of its effectiveness against non-microscopic discectomy, microdiscectomy and tubular discectomy. Spine J.

[34] Ruan W, Feng F, Liu Z, Xie J, Cai L, Ping A (2016). Comparison of percutaneous endoscopic lumbar discectomy versus open lumbar microdiscectomy for lumbar disc herniation: a meta-analysis. Int J Surg.

[35] Li X, Han Y, Di Z (2016). Percutaneous endoscopic lumbar discectomy for lumbar disc herniation. J Clin Neurosci.

[36] Kang TW, Park SY, Oh H, Lee SH, Park JH, Suh SW (2021). Risk of reoperation and infection after percutaneous endoscopic lumbar discectomy and open lumbar discectomy : a nationwide population-based study. Bone Joint J.

[37] Son S, Ahn Y, Lee SG, Kim WK (2020). Learning curve of percutaneous endoscopic interlaminar lumbar discectomy versus open lumbar microdiscectomy at the L5-S1 level. PLoS One.

[38] Nie H, Zeng J, Song Y (2016). Percutaneous endoscopic lumbar discectomy for L5-S1 disc herniation via an interlaminar approach versus a transforaminal approach: a prospective randomized controlled study with 2-year follow up. Spine (Phila Pa 1976).

[39] Chen Z, Wang X, Cui X, Zhang G, Xu J, Lian X (2022). Transforaminal versus interlaminar approach of full-endoscopic lumbar discectomy under local anesthesia for L5/S1 disc herniation: a randomized controlled trial. Pain Physician.

[40] He DW, Xu YJ, Chen WC (2021). Meta-analysis of the operative treatment of lumbar disc herniation via transforaminal percutaneous endoscopic discectomy versus interlaminar percutaneous endoscopic discectomy in randomized trials. Medicine (Baltimore).

[41] Wang D, Yang J, Liu C (2025). Comparative analysis of endoscopic discectomy for demanding lumbar disc herniation. Sci Rep.

[42] Yin J, Gao G, Chen S, Ma T, Nong L (2025). Comparative study between unilateral biportal endoscopic discectomy and percutaneous interlaminar endoscopic discectomy for the treatment of L5/S1 disc herniation. World Neurosurg.

[43] Chen Z, Zhang L, Dong J (2023). Percutaneous transforaminal endoscopic discectomy versus microendoscopic discectomy for lumbar disk herniation: five-year results of a randomized controlled trial. Spine (Phila Pa 1976).

[44] Chen Z, Zhang L, Dong J (2020). Percutaneous transforaminal endoscopic discectomy versus microendoscopic discectomy for lumbar disc herniation: two-year results of a randomized controlled trial. Spine (Phila Pa 1976).

[45] Chin BZ, Yong JH, Wang E, Sim SI, Lin S, Wu PH, Hey HW (2024). Full-endoscopic versus microscopic spinal decompression for lumbar spinal stenosis: a systematic review & meta-analysis. Spine J.

[46] Perez-Roman RJ, Gaztanaga W, Lu VM, Wang MY (2022). Endoscopic decompression for the treatment of lumbar spinal stenosis: an updated systematic review and meta-analysis. J Neurosurg Spine.

[47] Pairuchvej S, Muljadi JA, Ho JC, Arirachakaran A, Kongtharvonskul J (2020). Full-endoscopic (bi-portal or uni-portal) versus microscopic lumbar decompression laminectomy in patients with spinal stenosis: systematic review and meta-analysis. Eur J Orthop Surg Traumatol.

[48] Hwang YH, Kim JS, Chough CK (2024). Prospective comparative analysis of three types of decompressive surgery for lumbar central stenosis: conventional, full-endoscopic, and biportal endoscopic laminectomy. Sci Rep.

[49] Kotheeranurak V, Tangdamrongtham T, Lin GX (2023). Comparison of full-endoscopic and tubular-based microscopic decompression in patients with lumbar spinal stenosis: a randomized controlled trial. Eur Spine J.

[50] Wu PH, Chin BZ, Lee P (2023). Ambulatory uniportal versus biportal endoscopic unilateral laminotomy with bilateral decompression for lumbar spinal stenosis-cohort study using a prospective registry. Eur Spine J.

